# Case for Counsel

**Published:** 1889-05

**Authors:** George H. Clark


### CASE FOR COUNSEL.

A young woman, æt. twenty-four years, has been subject to dysmenorrhœa for several years. Her mother died of pneumonia, after inheriting a tuberculous tendency. Her father in his early manhood had rheumatism.

The menses are always preceded a day or two by pain of a sharp character in the lower abdomen. The pain is worse in the night, and she is sleepless. When the flow comes on the pain grows much worse, and is unendurable. It is a sharp, cutting pain, going from the front to the back of the pelvis, and running down the hips and thighs to the knees, and at times as far as the feet. During the pain she is obliged to sit up, as lying makes it much worse.

During the menses there is much heat and irritation in the vagina, particularly during the last two days, and the irritation gives rise to symptoms of nymphomania, but as she is very strong-willed, she is able to conquer the more pronounced symptoms of that condition. While this symptom is worse she cannot keep her legs still, but is rubbing them together constantly.

On the third or fourth day of the menses, she has paroxysms of opisthotonus, which usually come on about eight in the evening, and continues for two or three hours. Stramonium$^{2c}$ or $^{5c}$ is usually given for these paroxysms, and she then will be free until the next evening. This continues for three or four days, and does not reappear until the next menstrual period.

She is much depressed; very irritable. Everything seems unnatural; things and people seem so far off that the sensation of loneliness is terrible. This is during the first three or four days of the menstrual period. Before menses, dread of having the flow appear, because of the above. Feels as if she could not stand it. There is throbbing pain in top of head, drawing pain in back part that comes up from the back.

Head feels too full, and is hot internally. Across middle of top of head something pressing down hard, as if it would cut it. Soreness to touch over top of head. Wants to press head into something, but it hurts it to do so. Sharp pains all through head. Tight band around head. Trying to think makes all the symptoms worse, and she feels fairly wild. Head gets dull, feels perfectly blank. Bones in back of head feel knitted together.

Sharp pains through right eye, goes back into head, and at times goes down into cheek. When it is bad, there is a mist in front of the eye. Worse in high wind and in damp weather.

Ringing sound in ears; a far-off sound. Comes on when head is very bad. Almost deafening at times. Feeling of pressure; pressing out. Mouth gets very dry. Pain all through teeth; at times sharp, at others a dull aching. Bites tongue, cheeks, and lips; more when asleep. Throat is sore every night and morning. A sensation as of lump in throat; wants to swallow.

It worries her to have anything close about throat.

Soreness in region of stomach; cannot bear pressure about the waist.

Nausea that comes from head. Turning in stomach. Feels as if it were being stirred up.

Aching in abdomen, coming from the lower part, uterine region, and going through into back and down the legs.

This is constant, and is better from heat.

Dull, heavy pain in left ovarian region.

Feeling as if everything would be forced out of pelvis.

Abdomen very tender; cannot bear the least pressure.

Dull pain across upper part of abdomen.

Constipation. Hæmorrhoids, bleeding when bad; protrude.

Burning, throbbing pain in hæmorrhoids.

Soreness in left side of chest. Feels as if the ribs were running into her, or as if they were fast to something inside. K-ca.

Sharp pains in chest. Feeling of weight in chest, especially when falling asleep; makes her feel as though she could not breathe.

Burning, throbbing pain the entire length of the spine, which runs up into head. Clothing feels too warm on back.

Tenderness of spinal column.

Drawing in back and head, which, when bad, goes all through body, and she feels as if she could scarcely breathe. End of spine very tender.

Arms ache and feel too heavy. Sharp pain in right shoulder, goes down into hand. Numbness of arms and hands; they seem to go to sleep easily.

Legs ache, and feel heavy. Numbness of legs.

Sometimes feels unable to move arms or legs; she feels as if she had no power in them. Dragging in legs.

Feet perspire a great deal. Corns very sore. Coldness of feel, extending to above knees.

There is numbness all over, which comes from back.

Feeling as of prickly heat, all over; worse at night.

Feeling as if she could not move, as if all life had gone out of her; worse just before menses.

The sharp pains all through her are worse in damp weather.

Symptoms all worse before, during, and after menses, and when tired, and in damp weather. GEORGE H. CLARK.

